# 

**Published:** 1799-06

**Authors:** 


### *New Medical and Phyſical Publications, in May.*

ACCOUNT of the regular Gradation in Man, and in different animals and vegetables, and from the former to the latter, by *Charles White*. 4to. 10s. 6d. boards. Dilly.

Remarks on Mr. John Bell's Anatomy of the Heart and Arteries. 2s. 6d. Robinsons.

Practical Observations on the Diseases of the Army in Jamaica, as they occurred between the years 1792 and 1797. And on the means of lessening mortality among the troops, and among Europeans in Tropical climates. By *William Lempriere*, apothecary to the forces, 2 vols. 13s. boards. Longman and Rees.

Facts and Observations relative to the Nature and Origin of the Pestilential Fever, which prevailed in Philadelphia, in 1793, 1797, 1798. By the College of Physicians of Philadelphia. 9d. J. Phillips.

Further Observations on the Variolæ Vaccinæ, or Cow-pox. By *Edward Jenner*, M. D. 2s. 6d.

Reports of a Series of Inoculation for the Variolæ Vaccinæ, or Cow-pox; with Remarks and Observations on the Disease considered as a substitute for the small-pox. By *William Woodville*, M. D. &c. 8vo. 2s. 6d. Philips and Son.

An Essay on the most rational means of preserving Health. With Anecdotes of Longevity. 3s. boards. Wallis.

The Physician's Vade Mecum, by the Rev. *Joseph Townshend*. Fifth Edition. 4s. sewed. H. D. Symonds.

An Essay on the Causes, Early Signs, and Prevention of Pulmonary Consumption, for the use of Parents and Preceptors. By *Thomas Beddoes*. M. D. 5s. boards. Longman and Rees.

Advice to Commanders of His Majesty's Fleet serving in the West Indies, on the Preservation of the health of seamen, by *Leonard Gillespie*, M. D. Surgeon to the Naval Hospital, Fort Royal, Martinico. 1s. Cutheil.

Essays on the Venereal Disease and its concomitant affections: by *William Blair*, A. M. and first part of the first Essay. A new edition, corrected. Four Shillings. Johnson.

A De-

A Detection of the Fallacy of Dr. Hull's Defence of the Cæsarean Operation: by *W. Simmons* &c. 8vo. Manchester. 2s. 6d. Vernor and Hood.

The new Illustration of the Sexual System of Linæus. By *Robert John Thornton*, M.D. No. 1, to be continued every three months, and completed in 14 Numbers, 1l. 1s. each. H. D. Symonds.

NEW MEDICAL PUBLICATIONS IN GERMANY.

*Guter Rath an Muetter*, &c. Good Advice to Mothers, on the most important Points of the Physical Education of Children, in the first years of life. By Dr. C. W. HUFELAND, &c. 8vo. 86 pp. *Berlin.* Rottmann.

*Einige wohlgemeinte Vorschlæge*, &c. Some well intended Proposals for establishing a Medical College, on the most solid and perfect basis. (A satirical Oration.)

*Der Philosophische Arzt.*—The Medical Philosopher. By Dr. WEIKARD. Two Vols. 8vo.

—*Versuche*, &c.—Experiments on the irritability of Muscular and Nervous Fibres, Vol. II. 8vo. *Berlin.* Rottmann.

—*Anatomische Beschreibung*, &c.—An Anatomical Description of the external Parts of the human Eye, particularly of the lachrymal Vessels. Translated from the Latin of Dr. J. C. ROSENMULLER: with Additions and Improvements by the Author, 4to. with coloured Plates, (Price, in Sheets, 6 Dollars, Sax. Curr.) *Leipzig.* Linke.

*Abhandlung*, &c. A Treatise on various Diseases, which originally arise from Acrimony; such as various Cutaneous Affections, Scrophula, Syphilis. Tetters, Cancer and Gout; together with the Method of Cure, and the most approved Prescriptions. By Dr. J. V. MULLER. A new Edition, improved. *Leipzig.* Jaeger.

NEW MEDICAL PUBLICATIONS IN FRANCE.

*Extrait de la Partie Medicale des Memoires de l'Institut National*, &c.---An Exstract of the Medical Department of the Transactions of the National Institute for the fourth Year of the French Republic. Vol. I. No. 4. *Paris.* Baudoin.

*Traite des Maladies des Voies Urinaires*, &c.---A Treatise on the Disorders of the Urinary Passages. By P. J. DESAULT, extracted from the Journal of Surgery, enlarged and published by XAV. BICHAT. 8vo. *Paris*, Veuve Desault, *Cloitre Notre Dame.*

*Recherches sur quelques Points d'Histoire de la Medecine, &c.*---Inquiries respecting some Particulars of the History of Medicine, concerning Inoculation. By Citizen BOURDEAU, 12mo. 590 pp. (3 Livres 25 Cent.) *Paris.* Remont.

*Le Medecin des Goutteux*, &c. The Gouty Man's Physician. By L. BODIN, 8vo. (1 Livre 50 Cent.) *Paris.* Croullebois.

---

### ERRATA.

No. III. p. 303, line 10, after the words *last lecture*, add, *on Thursday, April 11th.*
——— p. 304, l. 18, for *Sennebteria*, read *Sennebieria.*
——— p. 309, l. 14, for *recourse*, read *resource.*
No. IV. p. 339, l. 12, for *life, and still permit poison in a thousand forms*, read *life;—that poison in a thousand forms should still be permitted.*
——— ibid. l. 24, after the word *traffic*, add, *the evil might be remedied.*

---

### TO CORRESPONDENTS.

WE have again been obliged to poſtpone ſeveral valuable papers, on account of their late arrival. We acknowledge the favours received, under the following ſignatures, viz. from T. P. of Alceſter; G. C. of Kidderminſter; W. V. of Rocheſter; C. B. of Hatton-Garden; and S. H. of the Strand:—if our room permit, they ſhall be inſerted in the next Number. We repeat our former requeſt, that Correſpondents may add the date of their letters, and their reſpective deſignations.

